# Nanocarrier-Mediated Targeting of Tumor and Tumor Vascular Cells Improves Uptake and Penetration of Drugs into Neuroblastoma

**DOI:** 10.3389/fonc.2013.00190

**Published:** 2013-08-05

**Authors:** Fabio Pastorino, Chiara Brignole, Monica Loi, Daniela Di Paolo, Annarita Di Fiore, Patrizia Perri, Gabriella Pagnan, Mirco Ponzoni

**Affiliations:** ^1^Experimental Therapy Unit, Laboratory of Oncology, Istituto Giannina Gaslini, Genoa, Italy

**Keywords:** drug delivery, targeting, nanocarriers, tumor vasculature, tumor uptake, tumor penetration, neuroblastoma

## Abstract

Neuroblastoma (NB) is the most common extracranial solid tumor in children, accounting for about 8% of childhood cancers. Despite aggressive treatment, patients suffering from high-risk NB have very poor 5-year overall survival rate, due to relapsed and/or treatment-resistant tumors. A further increase in therapeutic dose intensity is not feasible, because it will lead to prohibitive short-term and long-term toxicities. New approaches with targeted therapies may improve efficacy and decrease toxicity. The use of drug delivery systems allows site specific delivery of higher payload of active agents associated with lower systemic toxicity compared to the use of conventional (“free”) drugs. The possibility of imparting selectivity to the carriers to the cancer foci through the use of a targeting moiety (e.g., a peptide or an antibody) further enhances drug efficacy and safety. We have recently developed two strategies for increasing local concentration of anti-cancer agents, such as CpG-containing oligonucleotides, small interfering RNAs, and chemotherapeutics in NB. For doing that, we have used the monoclonal antibody anti-disialoganglioside (GD_2_), able to specifically recognize the NB tumor and the peptides containing NGR and CPRECES motifs, that selectively bind to the aminopeptidase N-expressing endothelial and the aminopeptidase A-expressing perivascular tumor cells, respectively. The review will focus on the use of tumor- and tumor vasculature-targeted nanocarriers to improve tumor targeting, uptake, and penetration of drugs in preclinical models of human NB.

## Introduction

Neuroblastoma (NB) is the most common solid tumor in children derived from the sympathetic nervous system and the commonest type of cancer to be diagnosed in the first year of life (1). The effective treatment of NB, either at advanced stages or at minimal residual disease, remains one of the major challenges in pediatric oncology. While Stage I and II tumors are localized and well differentiated, and can be successfully treated by surgical resection, patients with stage III and IV tumors present regional and disseminated disease with poor prognosis and low response to intensive therapeutic intervention and conventional treatments (2). Moreover, despite some advances, these tumors still have unacceptably low cure rates, and, even when treatment is successful, the acute and long-term morbidity of current therapy can be substantial (3, 4).

*In vitro* preclinical research has identified novel agents with promising therapeutic potential for the treatment of this malignancy, however their *in vivo* efficacy is limited by unfavorable pharmacokinetic properties resulting in either insufficient drug delivery and penetration into the tumor and/or metastatic sites, or high systemic and/or organ-specific toxicities.

Currently, anti-tumor compounds share, indeed, two properties: short half-life and small therapeutic index (the range of concentration between efficacy and toxicity). However, it has been demonstrated that the encapsulation of these “drugs” into nanocarriers drastically ameliorates their kinetic profiles, increasing tumor targeting and reducing side effects.

## Nanocarriers for Drug Delivery

The medical community has recently sought alternative therapies that improve selective toxicity against cancer cells, while decreasing side effects. Nano-biotechnology, defined as biomedical applications of nano-sized systems, is a rapidly developing area within nanotechnology (5). Nanoparticles, such as liposomes, allow unique interaction with biological systems at the molecular level. They can also facilitate important advances in detection, diagnosis, and treatment of human cancers and have led to a new discipline of nano-biotechnology, called nano-oncology. Nanoparticles are being actively developed for tumor imaging *in vivo*, biomolecular profiling of cancer biomarkers, and targeted drug delivery (6–8).

Growing solid tumors have capillaries with increased permeability as a result of the disease process (e.g., tumor angiogenesis). Pore diameters in these capillaries can range from 100 to 800 nm. Drug-containing liposomes that have diameters in the range of approximately 50–200 nm are small enough to extravasate from the blood into the tumor interstitial space through these pores (9). Normal tissues contain capillaries with tight junctions that are impermeable to liposomes and other particles of this diameter. This differential accumulation and penetration of liposomal drugs in tumor tissues relative to normal cells is the basis for the increased tumor specificity of liposomal drugs relative to free drugs. In addition, due to impaired and defective lymphatic vessels, tumors lack lymphatic drainage and therefore there is low clearance of the extravasated liposomes from tumors. Thus, this liposome-mediated passive targeting can result in increases in drug concentrations within solid tumors of several-fold relative to those obtained with free drugs. This phenomenon has been termed the enhanced permeability and retention effect (EPR) (10–12). This mechanism of action of the liposomal drugs is thought to be due to sustained release of drug from the liposomes and diffusion of the released drug throughout the tumor interstitial fluid, with subsequent uptake of the released drug by tumor cells.

At present, however, EPR effect has been measured mostly, if not exclusively, in implanted tumors with limited data on EPR in metastatic lesions. Moreover, EPR heterogeneity effect in different tumors (with either differences in vessel structures within a single tumor type, or different pore dimensions in the vasculature and changed pore size with the location for a given type of tumor) as well as limited experimental data from patients on the effectiveness of this mechanism, seems to hamper the progress in developing drugs using this approach (13). Furthermore, EPR effect has been demonstrated to be insufficiently performatory in different animal models of human NB used for testing our preclinical nanocarriers-based therapies (14–19), likely because of the above mentioned tumor heterogeneity.

Consequently, in the attempt of globally increasing the specificity of interaction of liposomal drugs with target cells and the penetration of more amount of drug delivered to latter, recent efforts in the nanocarriers field have been addressed to the development of Ligand-Targeted Liposomes (LTLs). These liposomes utilize targeting moieties coupled to the liposome surface, for delivering the drug-liposome package to the desired site of action (active targeting). Targeting moieties may include antibody molecules, or fragments thereof, small molecular weight naturally occurring or synthetic ligands like peptides, carbohydrates, glycoproteins, or receptor ligands, i.e., essentially any molecule that selectively recognizes and binds to target antigens or receptors over-expressed or selectively expressed on cancer cells (20).

The great advantages of LTLs encapsulating cytotoxic drugs over free drugs have been unquestionably demonstrated in a number of experimental models of cancer (15, 20–22). The mechanism whereby LTLs appears to act is related to the specific binding of the drug carrying liposomes to the selective receptor expressed on cell surface of tumor cells and the subsequent internalization of the liposomal drug package.

Interestingly, localized release of the encapsulated drug at the targeted cell surface may occur, contributing to the mechanism of drug penetration and cytotoxicity mainly due as a consequence of binding to the specific receptor(s) (11, 20). Since most tumors are heterogeneous with regard to tumor-associated-antigen expression, another advantage may be the “bystander effect”: specific binding of LTLs to a tumor cell, with release and diffusion of the drug into tumor parenchyma may result in cytotoxicity of bystander tumor cells lacking the specific epitope. It has been shown that approximately 400-fold more monoclonal antibody was required to achieve similar results with antibody-drug conjugates. Hence, high drug: antibody ratios can be achieved with LTLs, thus decreasing the need for expensive and potentially immunogenic antibodies.

## Tumor Cell Targeting Leads to Increased Uptake of Anti-Cancer Agents in NB

Neuroblastoma tumors express abundant amounts of the disialoganglioside GD_2_ at the cell surface and this epitope is only minimally expressed by normal tissues, such as the cerebellum and peripheral nerves (23), thus the use of anti-GD_2_ whole antibodies or their corresponding Fab′ fragments were used as a selective ligand for targeting liposomal “drug” to human NB cells.

Below are reported our recent results obtained by using “drug”-loaded, nanocarrier-mediated targeting of the GD_2_ epitope, via coupled anti-GD_2_ whole antibodies, with improved uptake and penetration of drugs into experimental models of human NB.

### Liposomal fenretinide

Due to the success of 13-*cis*-retinoic acid in NB high-risk patients with elevated frequency of relapse from minimal residual disease (24), an increased scientific interest has been consolidated in developing retinoids, a known class of molecules able to trigger both terminal differentiation and apoptosis/necrosis on NB cells (25, 26). In this *scenario*, newer chemotherapeutic approaches also count on the addition of more potent retinoids, such as fenretinide (HPR), a synthetic retinoic acid derivative, which has a very low degree of toxicity relative to others and has shown efficacy as a highly active and promising therapeutic and chemopreventive agent in different experimental models and clinical trials (27, 28). However, despite good tolerability in humans, therapeutic efficacy of HPR is limited by its relatively poor bioavailability particularly from ingested tablets (29). Indeed, the phase II study of oral capsular HPR has recently underlined how, this formulation is characterized by intraindividual and interindividual variation in pharmacokinetic features as HPR is too lipophilic to easily pass the intestinal membrane (30). This hindrance has prompted scientists to draw clinical protocols based on more appropriate HPR formulations with improved biodistribution after both oral route and intravenous injection and suitable also for pediatric use. Maurer et al. (31) has proposed a lipid complex to deliver HPR, called 4-HPR/Lym-X-Sorb (LXS), that was able to improve the retinoid solubility and oral bioavailability and to significantly increase plasma and tissue levels in mice (32). On the other hand, an *in vitro* study has proposed, as novel carriers for HPR, specific amphiphilic macromolecules formed by branched polyethylene glycol covalently linked with alkyl hydrocarbon chains: in this formulation, HPR is entrapped onto hydrophobic inner cores and the resultant complexes have dimensions suitable for intravenous administration (33).

**Figure 1:**
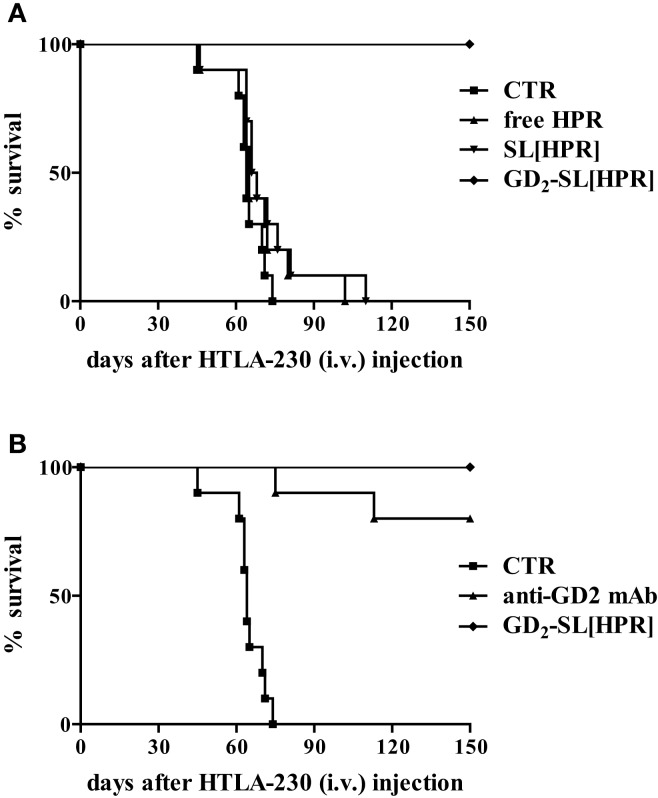
**Survival of neuroblastoma-bearing mice after treatment with fenretinide (HPR)-containing nanocarriers**. Nude mice were injected intravenously with 3 × 10^6^ HTLA-230 cells, and treated 4 h after with the following HPR formulations for 5 days: **(A)** Hepes Buffer pH 7.4, control (CTR); free HPR, 15 mg/kg/total dose; SL(HPR), 15 mg/kg/total dose; GD_2_-SL(HPR), 15 mg HPR/kg/total dose (containing 2 mg mAb/kg/total dose). In a second experiment **(B)**, a group of mice were treated with 2 mg of GD_2_ monoclonal antibody/kg/total (anti-GD_2_ mAb). All the experiments have been performed with *n* = 10 animals/group.

In order to improve tumor targeting, drug stability, and drug pharmacokinetics and bioavailability, we designed a formulation of HPR, encapsulated in sterically stabilized, GD_2_-targeted immunoliposomes [GD_2_-SIL(HPR)]. We demonstrated that HPR efficiently induced a dramatic inhibition of metastases, leading to almost 100% of curability in NB-bearing mice, only when encapsulated in GD_2_-targeted nanocarriers (14). These achievements totally disappeared when HPR was administered either free (free HPR) or loaded in non-targeted liposomes [SL(HPR)], confirming the importance of the tumor targeting as a mandatory tool for enhancing binding, uptake, and anti-tumor effects against NB (Figure 1A).

On the other hand, in this NB animal model, anti-GD_2_ monoclonal antibody (anti-GD_2_ mAb) also led to a considerable anti-tumor effect, indicating that the anti-GD_2_ “di *per se*” was responsible of part of the observed therapeutic effects (Figure 1B) (14). Thus, in the subsequent therapeutic, liposomes-based experiments we decided to use nanocarriers decorated with the non-immunogenic Fab′ fragments of anti-GD_2_, thus avoiding antibody-dependent cell cytotoxicity.

Indeed, the coupling of antibody Fab′ fragments instead of whole immunoglobulin molecules abolishes the mononuclear phagocyte system uptake of the anti-GD_2_, which takes place via an Fc receptor-mediated mechanism (34). Consequently, small LTLs decorated with Fab′ fragments have significantly longer circulation time than comparable formulations containing whole mAbs (20). This can result in an enhanced accumulation of the liposomes in solid tumor (35) and in a significant suppression of tumor growth (36, 37).

Here, we present some results obtained by using Fab′ fragments of anti-GD_2_ immunoliposomes to increase uptake and anti-tumor activity of CpG-containing antisense oligonucleotides (asODNs), small interfering RNAs (siRNAs), and chemotherapeutics in animal models of human NB.

### Liposomal antisense oligonucleotides

The identification of cancer-associated genes hold promise for the development of novel therapeutic strategies that selectively target tumor cells. asODNs can be used to specifically down modulate tumor associated gene expression (resulting in a direct anti-cancer effect) and as immune adjuvant by CpG-containing asODN-driven cytokines production and innate immune stimulation (38). However, since the *in vivo* applicability of ODNs is impaired by their high sensitivity to extracellular and cellular nuclease degradation (39), their encapsulation within liposomes should increase their stability. C-myb gene expression has been reported in several solid tumors of different embryonic origin, including NB, where it is linked to cell proliferation and/or differentiation (40, 41). We performed a new technique to encapsulate CpG-containing c-myb asODNs within lipid particles. Liposomes resulting from this technique were called coated cationic liposomes (CCLs) (41), since they were made up of a central core of a cationic phospholipids bound to myb asODNs and an outer shell of neutral lipids.

**Figure 2:**
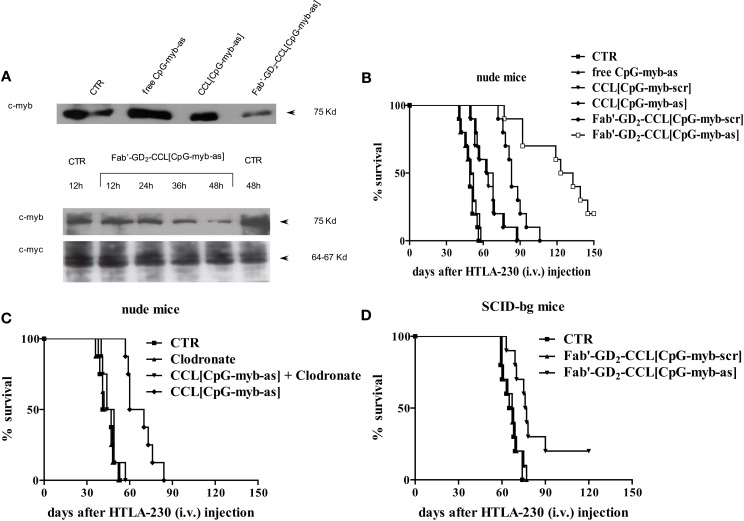
**Inhibition of c-Myb protein expression and increase of survival of neuroblastoma-bearing mice after treatment with c-myb antisense oligonucleotides (asODNs)-containing nanocarriers**. **(A)** GI-LI-N neuroblastoma cells were treated with CpG-containing c-myb asODNs, either free (free CpG-myb-as) or encapsulated in untargeted [CCL(CpG-myb-as)] and targeted [Fab′-GD_2_-CCL(CpG-myb-as)] nanocarriers, at a concentration of 100 μg/ml at the beginning of the experiment and 18 and 36 h later. Two hours after each addition, the cells were washed and transferred to CpG-myb-as-free fresh complete medium. The cells were harvested at 48 h (upper panel) or at the indicated time points (lower panels) and analysis of protein expression (c-Myb and c-Myc as control) was performed by immunoblotting. **(B)** Nude mice (*n* = 10 animals/group) were injected intravenously with 3.5 × 10^6^ HTLA-230 cells. Treatment with either as or scrambled (scr), CpG-containing ODNs, administered free and encapsulated in untargeted (CCL) and targeted (Fab′-GD_2_-CCL) liposomes was started at 4 h after cell inoculation. Mice were treated 4 days a week, for 2 weeks, with 3 day rest between treatments. Each mouse received 50 μg ODN. Control mice (CTR) received HEPES-buffered saline. **(C,D)** Effects of either macrophages or natural killer (NK) cells depletion on anti-tumor activity mediated by treatment with liposomal formulations containing ODNs. Mice [nude, *n* = 8 animals/group **(C)** and SCID-bg, *n* = 10 animals/group **(D)**] were inoculated with HTLA-230 cells and then treated as already mentioned in Figure 1. In some treatment groups of **(C)**, mice were injected with Clodronate-liposomes to deplete macrophages.

Fab′-GD_2_-targeted, CpG-containing c-myb asODNs-loaded CCLs reduced in a specific and time-dependent manner the expression of c-Myb protein by NB cells (Figure 2A). Importantly, we also demonstrated that their systemic administration in NB-bearing mice, induced anti-tumor effects with increased survival only when encapsulated in nanocarriers targeting the NB surface antigen, GD_2_, that internalizes after binding its ligand (Figure 2B) (17). We further demonstrated that increased animals life span was due to a dual mechanism of action. Firstly, a direct inhibition of tumor growth, via tumor cell binding, uptake, and inhibition of c-myb proto-oncogene expression; secondly, an indirect CpG-dependent immune stimulation, whose function was lost as the result of using clodronate-driven macrophage depletion in nude mice (Figure 2C) and B and NK cells depletion in SCID-bg mice (Figure 2D) (17).

### Liposomal small interfering RNAs

Despite the considerable potential of RNA interference (RNAi) for treating cancers (42, 43), several challenges still need to be overcome for exogenous siRNAs to be widely used as cancer therapeutics. The most significant hurdle is the specific and non-toxic delivery of siRNAs to the site of action. siRNA applications are so far limited almost to targets within the liver, where the delivery systems naturally occur, while delivery of siRNAs to extra-hepatic targets remain a serious challenge.

In order to solve this limitation, we consequently developed a new tumor-targeted delivery system for siRNAs, through their encapsulation into Fab′ fragments GD_2_-targeted coated CCLs, and validated their ability to silence the oncogene anaplastic lymphoma kinase (ALK) by increasing NB tumor binding and siRNA penetration-driven anti-tumor effect.

Indeed, over expression of either mutated or wild-type ALK tyrosine kinase receptor proteins induces constitutive kinase activity in NB (44), while ALK expression knockdown leads to a pronounced decreased cell proliferation. Moreover, ALK mutations and amplifications, as well as gene over expression, clearly correlate with poor outcomes in both advanced and metastatic NB disease, when compared with localized tumors (44). Based on these concepts, we tested the therapeutic efficacy of targeting ALK gene in NB, by developing a selective silencing approach (45).

**Figure 3:**
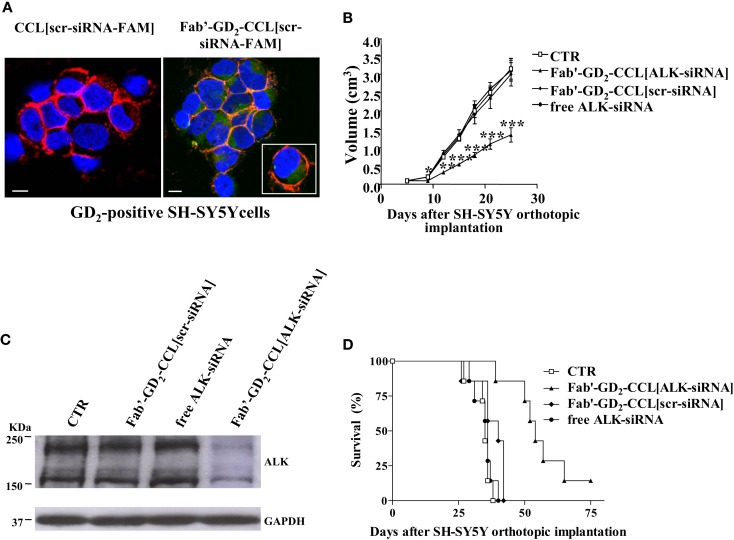
**Neuroblastoma-targeted nanoparticles entrapping siRNA specifically knockdown ALK**. **(A)** Uptake and internalization of liposome-encapsulated FAM-labeled scrambled-siRNA (scr-siRNA-FAM) into GD_2_-expressing neuroblastoma cells (SH-SY5Y). Cells were incubated at 37°C for 1 h, with either untargeted [CCL(scr-siRNA-FAM)] or Fab′ fragments GD_2_-targeted coated cationic liposomes [Fab′-GD_2_-CCL(scr-siRNA-FAM)]. After washing and cytospin centrifugation, cells were fixed and stained with a monoclonal antibody specific for the cellular adhesion molecule N-CAM (a-CD56) to reveal plasma membrane localization. Cell nuclei were stained with 4′,6-diamidino-2-phenylindole (DAPI). The cellular distribution of scr-siRNA-FAM (green), CD56 (red), nuclear DAPI staining (blue), and merged colors resulting from siRNA-liposome binding to the cell surface (orange) fluorescences was analyzed with a laser scanning spectral confocal microscope. Bars: 50 μm. **(B–D)** Tumor growth inhibition *in vivo* by ALK-siRNA encapsulated in Fab′-GD_2_-CCL. SCID-bg mice (*n* = 8) were orthotopically injected with 1.5 × 10^6^ SH-SY5Y cells in the capsule of the left adrenal gland. Seven days after the tumor implantation, mice were treated, two-time a week for 3 weeks with ALK-siRNA, either free or encapsulated in GD2-targeted nanocarriers [Fab′-GD_2_-CCL(ALK-siRNA)]. Another group of mice received a scrambled (scr) siRNA-loaded nanoparticles [Fab′-GD_2_-CCL (scr-siRNA)] as control or HEPES-buffered saline (CTR). Tumor expansion over time measured by calipers **(B)** and survival times **(D)** were used for determination of the treatment efficacy. **(C)** The day after the last treatment (25 day), tumors from three mice per group were recovered for western blot analyses and ALK protein expression. *P < 0.05; **P < 0.01; ***P < 0.001, Fab′-GD_2_-CCL(ALK-siRNA) vs. Fab′-GD_2_-CCL(scr-siRNA).

We showed that, while almost no binding and uptake was observed by siRNA-containing, untargeted liposomes [CCL(siRNA)] in NB cells, Fab′-GD_2_-targeted CCL(siRNA) were efficiently internalized (Figure 3A). Interestingly, in biologically relevant NB animal models, we demonstrated that, compared to free ALK-siRNA, Fab′-GD_2_-CCL(ALK-siRNA) increased siRNA stability, and a selective block of NB tumor growth, resulting in partial tumor regression (Figure 3B), improved silencing of the specific gene (Figure 3C), and increased life span in NB xenografts (Figure 3D) (45). This strategy also induced inhibition of angiogenesis and of metastatic potential in a safe and highly effective manner (46), confirming the pivotal role of targeted therapies to enhance tumor “drug” penetration and cytotoxic effects.

### Liposomal doxorubicin

To eradicate tumors with chemotherapy, anti-cancer drugs must reach lethal concentrations, in theory, in all of the tumor cells. Failure to achieve high local levels of drugs, e.g., due to limited drug delivery and/or penetration within tumors is critical for the effectiveness of solid-tumor chemotherapy (47). Methods for improving drug delivery and penetration in tumor tissues are, therefore, of great experimental and clinical interest. On this direction, one approach to selective eradicate NB tumor cells is based on the fact that NB is a chemosensitive tumor and cytotoxic agents, such as doxorubicin (DXR), are considered to be effective treatment modalities. However, the therapeutic efficacy of DXR, which is widely used in the treatment of solid tumors, is restricted by dose-limiting toxicity to bone marrow and heart tissue (48). The selective toxicity of DXR would be greatly improved if the concentration of drug in tumors could be increased relative to that in sensitive normal tissues.

Two strategies, based on tumor and vascular targeting, have been recently described for increasing the local concentration of the chemotherapeutic drug DXR in tumors and its therapeutic index. The first strategy is based on the direct targeting of the tumor cells by the use of Fab′ fragments of GD_2_-targeted DXR liposomal [Fab′-GD_2_-SIL(DXR)] (15). The second approach that will be discussed in the next chapter, is based on direct targeting of the tumor vasculature, using DXR encapsulated into modified liposomes able to bind and home to tumor blood vessels (16, 21, 49).

**Figure 4:**
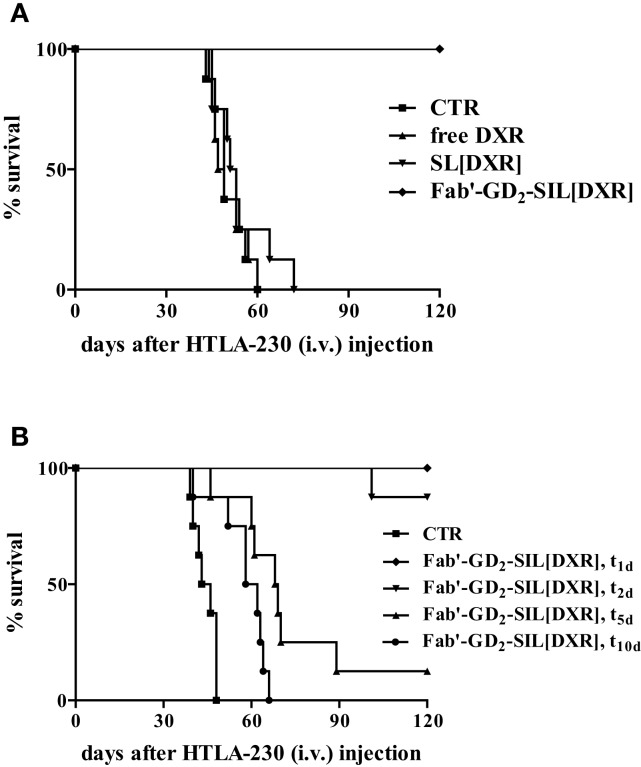
**Survival of neuroblastoma-bearing mice after treatment with doxorubicin (DXR)-containing, GD_2_-targeted nanocarriers**. **(A)** Nude mice received injections in the tail vein with 4 × 10^6^ HTLA-230 cells and 1 and 3 days post-inoculation mice received 5 mg/kg of DXR. Treatment groups (*n* = 8/group) consisted of DXR administered either free (free DXR) or encapsulated in untargeted [SL(DXR)] and targeted [Fab′-GD_2_-SIL(DXR)] nanocarriers. **(B)** Nude mice (8/group), inoculated i.v. with 4 × 10^6^ HTLA-230 cells, were treated on days 1, 2, 5, or 10 with 8 mg DXR/kg/week × 2 as Fab′-GD_2_-targeted nanocarriers [Fab′-GD2-SIL(DXR), t_1_, t_2_, t_5_, t_10_, respectively). Control mice (CTR) received HEPES-buffered saline. Partially reproduced from Pastorino et al. (21).

In the first study Fab′-GD_2_-SIL(DXR) has presented increased selectivity and efficacy in inhibiting NB cell proliferation compared to free drug and non-targeted DXR formulation. The *in vivo* anti-tumor activity of Fab′-GD_2_-SIL(DXR) was evaluated in terms of metastasis growth inhibition and increased life span in a pseudometastatic animal model of human NB. In this study, 100% of mice treated with DXR-loaded Fab′-immunoliposomes 1 day after tumor cells injection, were still alive more than 4 months after treatment. DXR administered either free or encapsulated in non-targeted nanocarriers did not show any anti-tumor effect, again confirming the important role of the specific tumor targeting in improving drug uptake and consequent tumor cells killing (Figure 4A) (15).

The next aim was to verify whether these anti-tumor effects were maintained in more established tumors or if the therapeutic efficacy declined when treatment was delayed. Indeed, a longer period of time between inoculation of cells and the administration of treatment would allow the tumor cells to establish metastases that might escape treatment. The metastatic cells would become less accessible from the vasculature and the tumor-targeted liposomes become less effective as their accessibility to the tumor cells becomes compromised. As expected, a delay in the start of treatment substantially reduced the therapeutic efficacy of Fab′-GD_2_-SIL(DXR), demonstrating the time dependence of the anti-tumor activity of the tumor-targeted formulation against advanced NB animal models (Figure 4B). However, with increasing time, the new lesions begin to recruit blood vessels to support their growth and the lesions will have increased sensitivity to anti-vascular therapy with time (21). Thus, our findings suggest the subsequent use of therapies targeting the vascular network of the tumor, as discussed below, to treat more mature solid tumors.

## Increasing Local Concentration of Anti-Cancer Agents in NB by Tumor Vasculature Targeting Strategy

The alternative strategy we pursued to increase the delivery and the uptake of DXR into NB is based on the use of tumor vasculature-targeted liposomes. The targeting of therapeutics to blood vessels, using probes that bind to specific molecular addresses in the vasculature, has indeed became a major research area (50). The inhibition of tumor growth by attacking the vascular supply of the tumor offers a primary target for therapeutic intervention. Indeed, host endothelial cells are believed to play a central role in tumor growth, progression, and metastasis, acting as the primary building blocks of the tumor microvasculature (51). Because of the “angiogenesis dependence” of solid tumors, predicted by Folkman nearly 40 years ago, selective inhibition or destruction of the tumor vasculature (using anti-angiogenic or anti-vascular treatments, respectively) could trigger tumor growth inhibition, regression, and/or a state of dormancy and thereby offer a novel approach to cancer treatment.

There are several advantages of targeting chemotherapeutic agents to proliferating endothelial cells in the tumor vasculature rather than directly to tumor cells. First, acquired drug resistance, resulting from genetic and epigenetic mechanisms reduces the effectiveness of available drugs (52). Anti-angiogenic/anti-vascular therapy has the potential to overcome these problems or reduce their impact. The tumor vasculature, composed of non-malignant cells that are genetically more stable than malignant cells, is therefore unlikely to mutate into drug-resistant variants. Second, the fact that a large number of cancer cells depend upon a small number of endothelial cells for their growth and survival might also amplify the therapeutic effect (53). Third, anti-angiogenic therapies may also circumvent what may be a major mechanism of intrinsic drug resistance, namely insufficient drug penetration into the interior of a tumor mass due to high interstitial pressure gradients within tumors (54). A strategy that targets both the tumor vasculature and the tumor cells themselves may be more effective than strategies that target only tumor vasculature, since this strategy can leave a cuff of unaffected tumor cells at the tumor periphery that can subsequently re-grow and kill the animals (55). Fourth, oxygen consumption by neoplastic and endothelial cells, along with poor oxygen perfusion, creates hypoxia within tumors. These pathophysiological characteristics of solid tumors compromise the delivery and effectiveness of conventional cytotoxic therapies as well as molecularly targeted therapies (53, 54). Finally, the therapeutic target is independent of the type of solid tumor; killing of proliferating endothelial cells in the tumor microenvironment can be effective against a variety of malignancies.

Phage display technology has been recently used to discover novel ligands specific to receptors on the surface of tumor epithelial and endothelial cells (56). *In vivo* panning of phage libraries in tumor-bearing mice has selected peptides that interact with proteins expressed on tumor-associated vessels and that home to neoplastic tissues (57). This technology, for instance, was used to isolate peptides homing specifically to the tumor blood vessel-associated addresses, aminopeptidase N (APN) and A (APA) (58, 59). We have firstly demonstrated that liposome-entrapped DXR, and targeted to APN via an NGR-containing peptide, induced tumor regression, pronounced destruction of the tumor vessels, and prolonged survival in NB-bearing mice (16).

**Figure 5:**
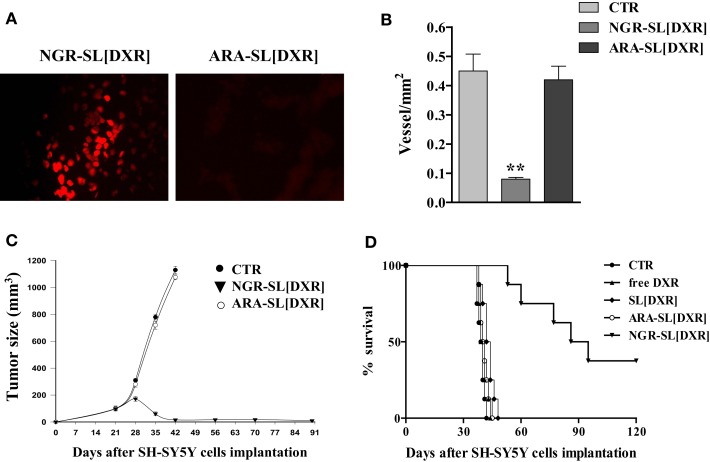
**Anti-angiogenic and anti-tumor effects of doxorubicin (DXR)-containing, tumor vascular targeted nanocarriers**. **(A)** Tumor homing of NGR-targeted liposomal DXR in SCID mice orthotopically injected in the left adrenal gland with 1.5 × 10^6^ SH-SY5Y neuroblastoma cells. DXR-loaded, either NGR-targeted or miss-matched peptide ARA-targeted nanocarriers, were injected via the tail vein as a single bolus dose. After 16 h, tumors were collected and DXR (red) visualized by fluorescence microscopy of fixed, paraffin embedded, tissue sections. **(B–D)** Delivery of DXR to tumor vessels inhibits angiogenesis, causing regression of established NB tumors. SCID mice orthotopically implanted with SH-SY5Y cells were injected intravenously with 3 mg DXR/kg, 21, 28, and 35 days post tumor inoculation. Treatment groups (*n* = 8/group) consisted of DXR administered either free (free DXR) or encapsulated in untargeted [SL(DXR)] and either NGR-targeted [NGR-SL(DXR)] or miss-matched peptide ARA-targeted [ARA-SL(DXR)] nanocarriers. Control mice (CTR) received HEPES-buffered saline. **(B)** Tumor vessels density inhibition after NGR-targeted liposomal DXR treatments. Orthotopic tumors, at day 36 from CTR and from both NGR- and ARA-targeted, DXR-treated groups, were sectioned and stained with an antibody to factor VIII to count blood vessels. Each bar represents the mean ± SD of five replicates. **(C)** Neuroblastoma tumor growth arrest by NGR-targeted liposomal DXR. Each point represents the mean ± SD of six replicates. **(D)** Increase in animal life span by NGR-targeted liposomal DXR. Partially reproduced from Pastorino et al. (16).

Specifically, to determine whether the NGR-targeted liposomes [NGR-SL(DXR)] could deliver DXR to angiogenic tumor-associated blood vessels, we injected them into the tail vein of mice bearing established adrenal tumors. In one set of experiments, liposomes were allowed to circulate from 2 to 24 h, followed by perfusion and immediate tissue recovery. There was a clear time-dependent uptake of DXR in the tumor vasculature. At 24 h, the staining pattern indicated that the DXR had spread outside the blood vessels and into the tumors (16). This spreading was attributable to increased permeability of tumor blood vessels to the intact liposomes (60) and/or uptake of the targeted liposomes by angiogenic endothelial cells and subsequent penetration and transfer to tumor cells. Likely both mechanisms are working at the same time. In the second set of experiments, tissues were examined 16 h after the injection of DXR-loaded liposomes, decorated with either the specific NGR [NGR-SL(DXR)] or with the miss-matched ARA [ARA-SL(DXR)] peptides. Strong DXR staining in tumor vasculature was seen only in animals treated with NGR-liposomes (Figure 5A). Tumor-specific DXR uptake was completely blocked when mice were co-injected with a 50-fold molar excess of the soluble NGR peptide (16), confirming the peptide recognizing tumor vasculature-driven cell drug binding and penetration.

Histopathological analysis of cryosections taken from NGR-SL(DXR) treated mice revealed pronounced destruction of the tumor vasculature. Indeed, double staining of tumors with TUNEL and anti-factor VIII antibody or anti-human NB, demonstrated endothelial cell apoptosis in the vasculature as well as increased tumor cell apoptosis (16). Moreover, mice displayed rapid tumor regression, inhibition of metastases growth, and suppression of blood vessel density, while mice treated with ARA-SL(DXR) formed large well-vascularized tumors (Figures 5B–D) (16).

Subsequently, we developed a novel liposomal formulation targeting the perivascular tumor cell marker APA, expressed in the vascular wall of NB primary and metastatic lesions.

The primary goal of this study was to validate the hypothesis that the combined targeting of both the tumor endothelial cells (recognizing APN) and the pericytes (recognizing APA), supporting the vessels wall within the tumor, has improved tumor targeting, uptake, drug penetration, and therapeutic effects relative to each therapy alone.

**Figure 6:**
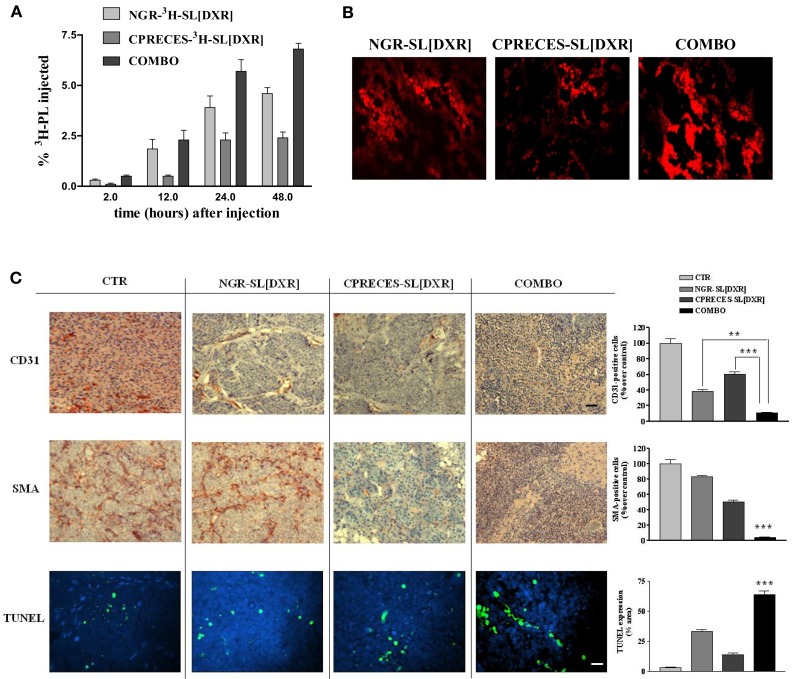
**Combined targeting of endothelial and perivascular tumor cells enhances anti-tumor efficacy of liposomal doxorubicin (DXR) in neuroblastoma**. **(A,B)** Accumulation of APN- and APA-targeted, DXR-loaded, nanocarriers in nude mice orthotopically implanted with GI-LI-N neuroblastoma cells. **(A)** ^3^H-labeled, endothelial- (via NGR peptide) and perivascular- (via CPRECES peptide) targeted, DXR-loaded nanocarriers were injected intravenously in a single bolus. Treatment groups consisted of NGR-^3^H-SL(DXR), CPRECES-^3^H-SL(DXR), and combination of targeted liposomes (COMBO). At selected time points post-injection, blood was measured for ^3^H in a beta-counter. Points, average of three mice; bars, ±95% C.I. **(B)** Tumor accumulation of DXR visualized by fluorescence microscopy of NB tissue sections. Magnitude, 40×. **(C)** Effects of the combination therapy on endothelial, perivascular, and tumor cells *in vivo*. Immunohistochemistry was performed on established NB tumors removed from untreated mice (CTR) or from mice treated with DXR-loaded, NGR-targeted or CPRECES-targeted nanocarriers, or with a combination of the two liposomal formulations (COMBO). Tumors were removed on day 36 and tissue sections were immunostained for CD31 and SMA to detect tumor vasculature (scale bar, 250 μm). TUNEL was performed to detect tumor apoptosis (scale bar, 100 μm). Cell nuclei were stained with DAPI. Columns, mean of CD31, SMA, and TUNEL staining intensities; error bars represent 95% C.I. **P < 0.01; ***P < 0.001, COMBO vs. single treatments.

Neuroblastoma-bearing mice receiving APA-targeted liposomal DXR [CPRECES-SL(DXR)] exhibited an increased life span in comparison to control mice, but to a lesser extent relative to that in mice treated with APN-targeted formulation [NGR-SL(DXR)] (18). However, mice treated with a combination (COMBO) of APA- and APN-targeted, liposomal DXR had an enhanced accumulation of both the carriers and the drug in the tumor mass (Figures 6A,B), and a significant increase in life span compared to each treatment administered separately (18). There was a significant increase in the level of apoptosis in the tumors of mice on the combination therapy, and a pronounced destruction of the tumor vasculature with nearly total ablation of endothelial cells and pericytes (Figure 6C). Thus, these results clearly demonstrated that the combined targeted strategy, through an increased drug penetration, was more effective for destruction of the tumor vasculature than either monotherapy. Combination therapy led to a statistically significant increase in life span in a murine xenograft model of human NB compared to the formulations given alone (18).

## Concluding Remarks

In a tumor mass, neoplastic cells and the vascular endothelium of angiogenic blood vessels that support tumor growth express targetable surface markers that are accessible from the circulation. Thus, targeting therapeutic agents to tumor cells and to tumor vessels made it possible to deliver the anti-cancer agents to the tumor site, and to combine blood vessel destruction with the conventional anti-tumor actions of drugs, leading to more efficacious effects and less systemic toxicity than conventional therapy.

However, despite good results obtained in preclinical experimental models, targeted therapies have also practically met with some drawbacks, restricting their clinical translation. In particular, this approach has only a partial success for the treatment of well-established solid tumors, where tumor vessels are poorly perfused with blood and are dysfunctional, limiting the delivery of blood-borne compounds into the tumor masses (61). Tumors have also an high interstitial pressure deriving from dysfunctional lymphatics, which causes tissue fluid to flow out of the tumor thus reducing diffusion of drugs from the blood vessels into the tumors (61, 62). Finally, interstitial fibrosis can further retard the diffusion of targeted compounds through the dense tumor parenchyma (63).

Consequently, to further overcome these drawbacks and to increase anti-tumor efficacy of the targeted therapies, in the near future the use of targeting probes with even more enhanced tumor-penetrating properties and receptors that are likely shared between tumor vessels and tumor cells should be envisaged.

## Conflict of Interest Statement

The authors declare that the research was conducted in the absence of any commercial or financial relationships that could be construed as a potential conflict of interest.
